# External defibrillation resulting in loss of ventricular capture during atrial lead testing

**DOI:** 10.1016/j.hrcr.2021.11.018

**Published:** 2021-11-27

**Authors:** Nicholas Palmeri, Andrew Locke, Patricia Tung

**Affiliations:** Beth Israel Deaconess Medical Center, Boston, Massachusetts

**Keywords:** Capture threshold, Device troubleshooting, External defibrillation, Lead management, Pacing

## Introduction

Adverse effects of external cardioversion on lead function and battery life of implanted cardiac devices have been previously described. More recent data have demonstrated that these effects are less common with the use of biphasic energy and bipolar leads.^1^ The safety data with contemporary devices is based on external cardioversion for atrial arrhythmias, and less is known about effects on device function after defibrillation for ventricular arrhythmias. Here we describe a case of right ventricle (RV) pacing lead failure following external defibrillation discovered during atrial lead testing and subsequent management.

## Case report

**Figure 1:**
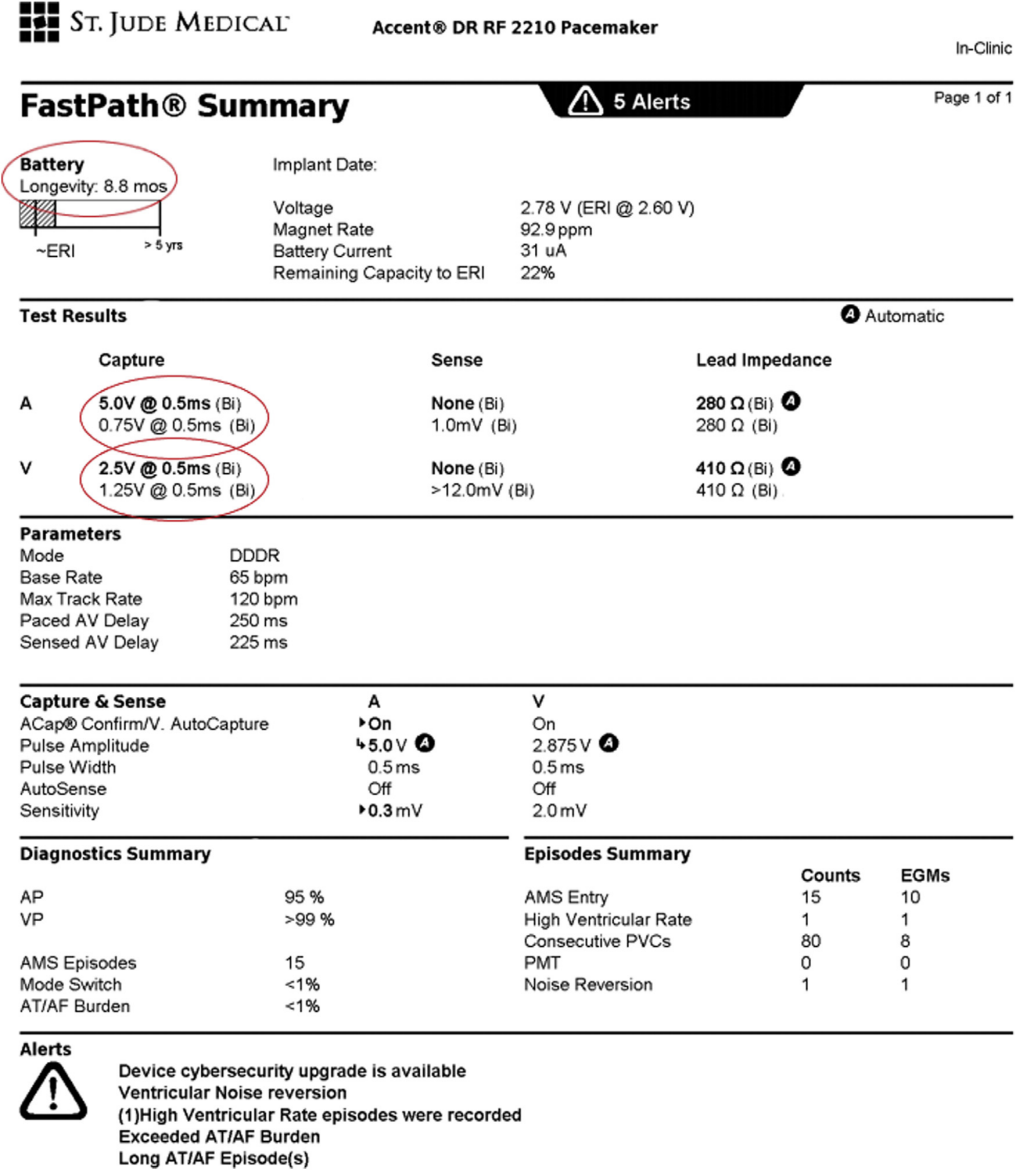
Initial interrogation. Increased atrial and ventricular capture thresholds were observed, as well as decreased battery voltage. (Note: the image has been minimally modified to exclude identifying patient information and dates.)

**Figure 2:**
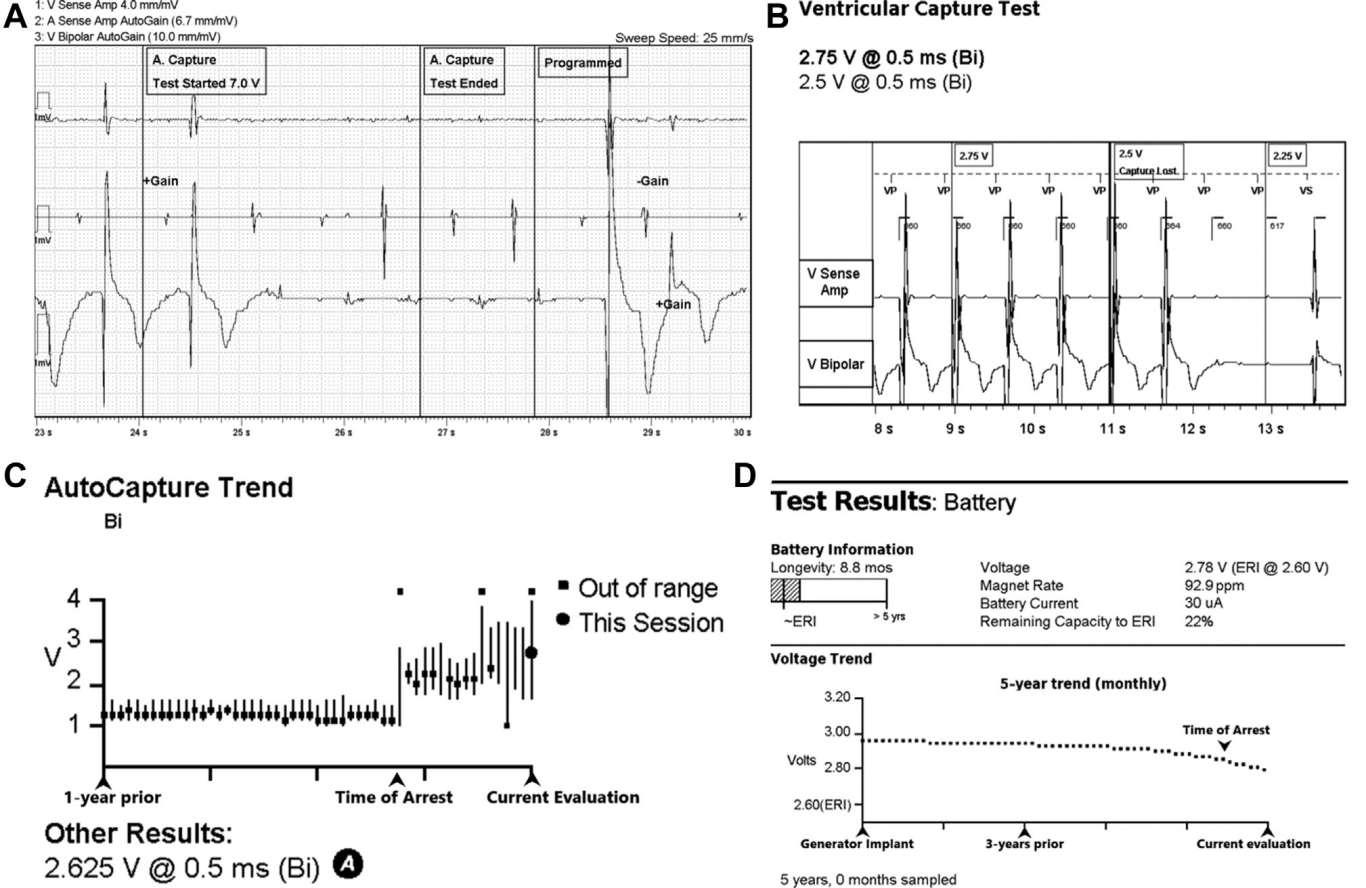
Device testing. **A:** Atrial lead testing caused loss of right ventricle (RV) capture. **B:** Reliable RV lead capture in VVI. **C,D:** Increase in RV capture threshold and decline in battery life beginning 6 months prior.

A 94-year-old woman with hypertension and paroxysmal atrial fibrillation had undergone implantation of a right-sided dual-chamber pacemaker at another hospital 13 years earlier for sinus node and atrioventricular conduction disease. Over time, she developed atrial and ventricular pacemaker dependence. She subsequently underwent a generator change 7 years after initial implant. She had been lost to follow-up and was not enrolled in remote monitoring when she presented to our outpatient clinic to reestablish care after an interval of more than 2 years. She denied any history of syncope or presyncope and her 12-lead electrocardiogram demonstrated A-V sequential pacing. Initial interrogation was notable for a remaining battery life of 8.8 months, which was decreased from 6 years at her visit 2 years earlier. Summary interrogation was also notable for elevated atrial and RV pacing thresholds compared to her prior interrogation (Figure 1). Atrial threshold testing in DDD mode repeatedly resulted in ventricular loss of capture associated with lightheadedness despite reliable capture of the RV lead in VVI mode (Figure 2A and 2B). The atrial threshold could not be confirmed owing to this issue. Ventricular capture threshold testing in VVI mode confirmed an increased threshold of 2.5 V @ 0.5 ms with stable lead impedance. Review of the pacemaker diagnostics revealed an abrupt increase in the RV pacing threshold beginning 6 months prior to the current evaluation with a corresponding decrease in battery life (Figure 2C and 2D).

**Figure 3:**
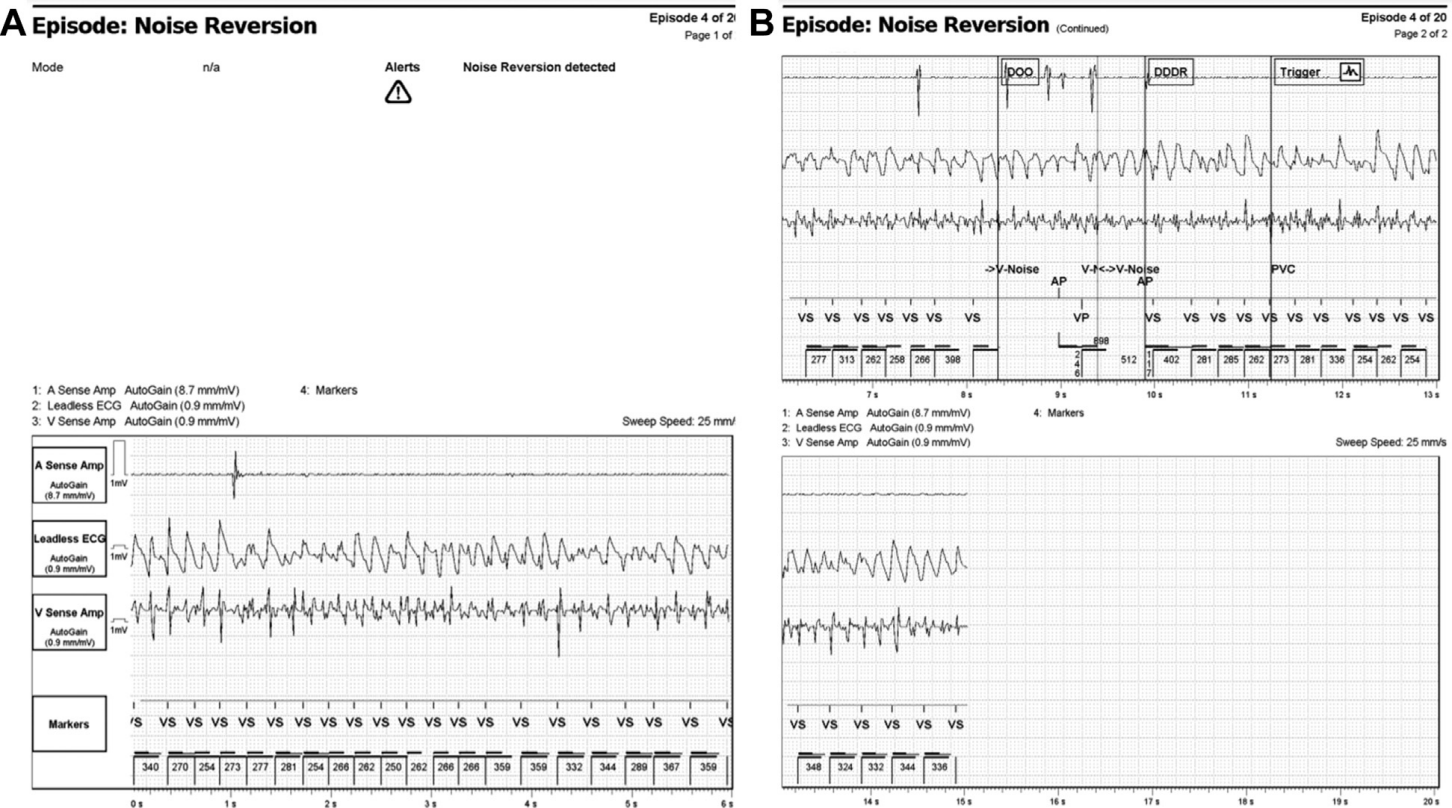
Noise reversion episode. Ventricular fibrillation, characterized as noise reversion, for which the patient was externally defibrillated.

On further review of the device diagnostics, an episode denoted as noise reversion demonstrated ventricular fibrillation, which occurred 6 months prior to the current evaluation (Figure 3). On further questioning, the patient confirmed an episode of acute chest pain, for which emergency medical services were called, at that time. Inferior ST-elevation elevations were documented with subsequent ventricular fibrillation and witnessed cardiac arrest. A single external shock was delivered, with return of spontaneous circulation. She was taken to another hospital where she had 2 drug-eluting stents placed in the right coronary artery. Following a complicated hospital course, she ultimately regained her baseline neurologic status and was discharged. An echocardiogram demonstrated an unchanged low-normal ejection fraction.

Though she had an estimated 8 months of battery life remaining and appeared to have reliable, though elevated, capture of the RV lead, the patient was scheduled for urgent implantation of a new RV pacing lead, possible insertion of a new atrial lead, and generator change, given her pacemaker dependence and unclear function of the chronic leads. Upon arrival to the electrophysiology lab, repeat interrogation yielded the same results as in clinic. After discussion with the manufacturer’s technical services department, concern for subthreshold ventricular output during atrial threshold testing was raised. We then disabled the ventricular autocapture algorithm, and threshold testing of the atrial and ventricular leads was successfully completed and demonstrated stable atrial capture threshold, impedance, and sensing, which was unchanged from prior. Atrial lead testing did not result in ventricular loss of capture and the RV pacing threshold was confirmed to be stably elevated at 2.875 V @ 0.4 ms with unchanged impedance. Given these findings, the existing leads were left in place and a generator change alone was performed. These results were confirmed via direct lead testing after disconnection from the device during the procedure.

The patient was evaluated at 1 week and at 3 months following the generator change and was found to have normal sensing and impedances and a stable ventricular pacing threshold and unremarkable testing with the autocapture algorithm enabled on her new generator.

## Discussion

Here we describe loss of ventricular capture during atrial lead testing in a patient with chronic pacing leads following external defibrillation for ventricular fibrillation. Analysis of the explanted generator by the manufacturer’s technical services department confirmed a previously unknown flaw in which the ventricular output reverts to the last programmed output during manual testing of the atrial lead when the autocapture algorithm is enabled. In this patient’s case, her ventricular threshold had increased, likely as a result of external defibrillation, and thus her last programmed ventricular output was inadequate and resulted in subthreshold stimulation and loss of capture associated with presyncope during testing. This malfunction is believed to be limited to this particular pacemaker model and limited to isolated atrial lead testing as was performed in clinic, such that this would not occur during autocapture testing of both leads while the patient was at home. The RV pacing threshold was elevated following external defibrillation, which accelerated battery depletion. These findings likely occurred owing to placement of the external defibrillation pads in close proximity to the right-sided device, leading to damage to the pacemaker circuitry and resulting in high-output ventricular pacing and accelerated battery depletion. It is possible that with proper placement of the defibrillation pads, further from the device site, damage to the pacemaker circuitry could have been avoided.

External cardioversion is routinely performed in patients with implanted cardiac devices, most commonly for atrial arrhythmias, and existing literature suggests that this is safe.^2^^,^^3^ There is scant literature on external defibrillation for ventricular arrhythmias on permanent pacemakers.^4^ Defibrillation in a right-sided pacemaker resulting in loss of capture has been reported, but on autopsy this seemed to be related to local RV infarction, presumably due to thermal injury from an induced current and use of unipolar leads.^5^ In this case, direct testing of the RV lead demonstrated consistent capture. Though thermal injury to the local RV myocardium from induced current during the shock may have contributed to accelerated battery depletion by increasing the pacing threshold, compromise of the pulse generator circuitry also accelerated battery depletion.

In this case, given the chronicity of the leads and the patient’s age, lead extraction was not considered. Based on the initial findings in clinic, placement of a new ventricular lead and possibly a new atrial lead along with generator change was recommended. Given the patient’s advanced age, extensive discussions were held with the patient and her caregivers and ultimately, she agreed to proceed. After discovery of the autocapture anomaly described above, lead stability was confirmed and the patient underwent generator change alone and was discharged following the procedure. Nearly 6 months following the generator change, the device battery was adequate, with stable lead parameters. The patient was successfully enrolled in remote monitoring to ensure ongoing surveillance of device function.

## Conclusions

Here, we describe loss of capture of a chronic RV pacing lead during atrial lead testing following external defibrillation in a patient with a right-sided pacemaker, which resolved with disabling of the autocapture algorithm. This resulted from a previously unknown malfunction in which the ventricular output reverts to the last programmed output rather than the autocapture value during manual testing of the atrial lead. In this case, we suspect the increased ventricular pacing threshold resulted from external defibrillation. Fortunately, this patient did not experience any adverse event despite not being enrolled in remote monitoring or having regular device follow-up. As a result of the discovery of this malfunction, she was able to undergo generator change alone and did not require the additional morbidity of lead extraction or placement of additional leads. Despite existing literature suggesting that external defibrillation and cardioversion is safe in patients with permanent pacemakers, close follow-up is necessary to ensure appropriate device function.

**Key Teaching Points**

• External defibrillation has the potential to damage pacemaker circuitry, particularly in right-sided devices.
• Complex circuitry employed in autocapture algorithms may malfunction and result in loss of capture despite a functional pacemaker lead.
• These changes may accelerate battery depletion, necessitating early generator change.
